# Selection of Level-Dependent Hearing Protectors for Use in An Indoor Shooting Range

**DOI:** 10.3390/ijerph16132266

**Published:** 2019-06-27

**Authors:** Rafal Mlynski, Emil Kozlowski

**Affiliations:** Department of Vibroacoustic Hazards, Central Institute for Labour Protection–National Research Institute, Czerniakowska 16, 00-701 Warsaw, Poland; emkoz@ciop.pl

**Keywords:** impulse noise, occupational exposure, hearing protectors, noise measurement, sound pressure level, level-dependent earmuffs, level-dependent earplugs

## Abstract

The high sound pressure level generated by impulse noise produced in an indoor shooting range makes it necessary to protect the hearing of the people it affects. Due to the need for verbal communication during training at a shooting range, level-dependent hearing protectors are useful. The objective of this study was to answer the question of whether it is possible to properly protect the hearing of a shooting instructor using level-dependent hearing protectors. The noise parameters were measured in the places where the instructor was present at the shooting range. The division of a specific group of trained shooters into subgroups consisting of three or six simultaneously shooting individuals did not significantly affect the exposure of the shooting instructor to the noise. An assessment of noise reduction was carried out for eight models of earmuffs and two variants of earplugs, using computational methods for the selection of hearing protectors. Among the noise parameters, both the A-weighted equivalent sound pressure level and the C-weighted peak sound pressure level were taken into account. Depending on the assessment criterion adopted, a sufficient reduction in impulse noise was provided by either four or six out of the 10 hearing protectors included in the study.

## 1. Introduction

Impulse noise is particularly dangerous for hearing due to its fast-changing nature. A single exposure to noise produced from an artillery shot can lead to a temporary hearing loss, while repeated exposure to noise associated with gunfire can result in permanent noise-induced hearing loss [1]. Auditory dysfunction after the blast results from the loss of outer hair cells and decreased spiral ganglion neurons and afferent nerve synapses [2]. Studies also showed that exposure to impulse noise caused a loss about 30% of inner hair cell synaptic ribbons in rat cochleae and the loss of about 10% of outer hair cells [3]. The occurrence of impulse noise is very often associated with the inability to apply engineering measures or administrative methods to reduce noise, which is especially true for people who are inside shooting ranges, where the sound source is in close proximity to the people. In contrast to sources of impulse noise in industry, there is no possibility to replace people with devices on a shooting range. The only known technical method of reducing the noise produced by firearms is the use of firearm suppressors [4]. It has been found that the effectiveness of suppressors can significantly exceed the noise reduction of hearing protectors [5]. However, this effectiveness, at a level of 20–28 dB, was also determined as not always sufficient [4]. In one of the reported studies, it was found that, despite the fact that suppressors significantly lower the peak sound pressure level (SPL) produced during shots—e.g., from AR-15 rifles—in most cases, its value exceeds 140 dB [6]. Due to the fact that the values of parameters of noise generated by shots most often exceed those that may be harmful to hearing, it becomes necessary to apply the latter possible solution, namely hearing protectors. The need to use hearing protectors is highlighted by the negative effects of the lack of such protection during the exposure of military personnel to noise from rifle shots [7]. The average threshold shift of soldiers exposed to noise produced by rifle shots was over 21 dB [7]. A recommendation for the use of hearing protectors was also formulated in light of conclusions from studies carried out among military personnel which suggested that even exposure to noise levels lower than the exposure limit value may lead to acoustic trauma [8]. Recommendations were also made that firearm users should always wear hearing protectors when shooting or hunting, since the use of firearm suppressors may not be sufficiently effective [4]. Re-educating people about the importance of proper hearing protection was also a result of research which found, among other things, that the risk of high-frequency hearing loss, e.g., in men involved in hunting, increased by 7% for every 5 years of participation in this type of activities [9].

The need for communication in the presence of noise, in terms of the use of hearing protectors, is becoming increasingly common in many workplaces [10]. For noise with an SPL that changes over time, it is advisable to use level-dependent hearing protectors. The level-dependent function is carried out by means of electronic circuits. In moments of relative silence, this function supports verbal communication and the possible hearing of other non-speech sounds. This does not require the removal of hearing protectors, which is beneficial on the one hand, and protects against randomly occurring sounds on the other. This functionality is particularly valuable at shooting ranges, because a person who wears hearing protectors at the shooting range is not exposed to the accidental acoustic impulse created by firing a firearm.

Regardless of the aforementioned functionality of level-dependent hearing protectors in terms of support in the perception of ambient sounds, these protectors should limit exposure to noise in such a way that the hearing of their users is safe. Therefore, it is necessary to properly select hearing protectors on the basis of the noise parameters present in the location of the user of the hearing protectors. The selection of hearing protectors can be carried out using computational methods. It is then necessary to determine both the noise parameters present in the workplace and information about the sound attenuation of the hearing protector in question. The selection of hearing protectors is not often discussed and, for example, is limited to presenting the basic principles of such a selection or discussing the main problems involved [10,11]. It is also mentioned that one of the problems in the assessment of the effectiveness of using hearing protectors is when noise is impulsive [11].

It is not difficult to obtain information about the sound attenuation of hearing protectors used in computational selection methods for continuous noise. This attenuation is one of the parameters measured during the conformity assessment process for the EU regulation [12], and its values are included in the user instructions for the hearing protectors. Some difficulties are related to the assessment of impulse noise parameters. The selection of hearing protectors requires precise data on the noise parameters, and the information that the exposure limit values of these parameters have been exceeded is inadequate. Due to the high SPL values of impulse noise exceeding the upper limits of the measurement range of standard sound level meters, measurements of noise parameters must be carried out with the use of appropriate equipment. The experience of research personnel is also crucial to properly determine representative cycles of tasks when determining noise parameters, as required by the test methodology [13].

The objective of this study was to answer the question of whether it is possible to properly protect the hearing of a shooting instructor at an indoor shooting range, using level-dependent hearing protectors. The study includes a large number (10) of level-dependent hearing protectors. In addition, the assessment of impulse noise reduction was carried out not in the presence of laboratory-produced acoustic impulses, as in the method described in ANSI/ASA S12.42-2010 (The American National Standards Institute, Inc./The Acoustical Society of America) [14], but instead measurements of noise parameters were carried out in a real acoustic environment at an indoor shooting range. During the tests, a full program of shooting was carried out using various types of weapons and ammunition. The only known example of a study referring to the possibility of reducing the exposure to noise at an instructor’s location is a study which concerned the use of suppressors rather than hearing protectors [4].

## 2. Materials and Methods

### 2.1. Hearing Protectors Included in the Assessment

Level-dependent hearing protectors were included in the assessment of the protection of the hearing of a shooting instructor. There were eight different models of earmuffs and two variants of earplugs with two types of eartips made of polyurethane and silicone, respectively. The use of the two types of eartips is associated with different sound attenuation values. Therefore, in this study, both variants of eartips are considered as two separate hearing protectors. In Table 1, the sound attenuation data provided in the user instructions for individual hearing protectors are presented, which are determined in accordance with the principles set out in EN ISO 4869-1 [15]. Symbols HP1 to HP7 and HP10 (HP—hearing protector; designations adopted in this study) denote level-dependent earmuffs. Symbols HP8 and HP9 apply to earplugs. Data from Table 1 were used in the selection of the hearing protectors.

### 2.2. Locations of Noise Parameter Measurements

**Figure 1:**
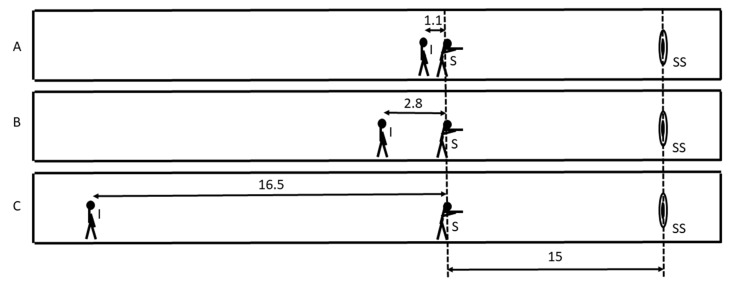
The three measuring situations (A, B, C) included in the measurement of noise parameters carried out at the indoor shooting range: I—shooting instructor, S—shooters, and SS—shooting target. Distances are expressed in meters.

Measurements of noise parameters were carried out during shooting exercises at the acoustically treated indoor shooting range, the dimensions of which were 35 m in length, 8.5 m in width and 2.6 m in height. As the possibility of protecting the shooting instructor’s hearing was being assessed, noise measurements were carried out in the places where the shooting instructor was present during the training, with the instructor at three different distances from the shooters: 1.1 m, 2.8 m, and 16.5 m (Figure 1).

### 2.3. Type of Weapon/Ammunition

The measurements were carried out during training, which consisted of four types of shooting cycles, during which shots were fired with the following weapon/ammunition combinations:

- Glock 17 pistol (9 × 19 mm Parabellum calibre) and Walther P99 (9 × 19 mm Parabellum calibre), hereinafter referred to as the ‘pistols’;
- PM-98 Glauberyt submachine gun (9 × 19 mm Parabellum calibre), hereinafter referred to as the ‘submachine gun’;
- Mossberg 590 smooth-bore shotgun (12/70 calibre: flash-bang ammunition and rubber ammunition), hereafter referred to as ‘shotgun 1’;
- Mossberg 590 smooth-bore shotgun (12/70 calibre: slug load ammunition and buckshot ammunition), hereafter referred to as ‘shotgun 2’.

### 2.4. Measurement Method

The measurements included a total of 53 shooting cycles, of which 17 cycles were shot from pistols, 16 cycles were related to shots from a submachine gun, 10 cycles were shot from (smooth-bore) shotgun 1 and 10 next cycles were shot from (smooth-bore) shotgun 2.

The training took place with two variants of the number of shooters simultaneously shooting during the shooting cycle: three or six shooters. The duration of one shooting cycle was 300 s (three shooters) or 420 s (six shooters). The duration of one shooting cycle means a full representative cycle of the task, determined in accordance with the principles for noise measurements at the workplace specified in the standard EN ISO 9612 [13]. The cycle at the shooting range included actions preceding shooting—i.e., giving instructions, loading weapons and other preparatory activities; shooting; and actions after shooting related to unloading weapons, checking their condition, etc.

The following noise parameters were determined as necessary for the assessment of noise reduction by hearing protectors:

- SPL in octave bands with center frequencies: 125 Hz, 250 Hz, 500 Hz, 1000 Hz, 2000 Hz, 4000 Hz, 8000 Hz.
- A-weighted equivalent SPL (L_Aeq_),
- C-weighted equivalent SPL (L_Ceq_),
- C-weighted peak SPL (L_Cpeak_).

Due to the fact that, in the assessment of noise exposure at the workplace, both the parameter related to the impulse nature of noise, L_Cpeak_, and the parameter reflecting the energy properties of noise, i.e., the A-weighted noise exposure level normalized to an 8-h working day (otherwise also expressed as daily noise exposure level (L_EX,8h_) [18,19]), are taken into account, both of these parameters were considered in this study. The determination of L_EX,8h_ is based on L_Aeq_, taking into account the time of exposure to noise $T_{e}$ (expressed in seconds), according to Equation (1) from the standard EN ISO 9612 [13].

(1) $$L_{\mathrm{EX},8h}=L_{\mathrm{Aeq}}+10\times \log_{10}\left(\frac{T_{e}}{8\times 60\times 60}\right)$$

### 2.5. Criteria for Assessing Exposure to Noise at the Workplace

Noise is classified as dangerous to hearing when an exposure limit value of one of the parameters, i.e., L_Cpeak_ or L_EX,8h_, is exceeded. For the L_Cpeak_ parameter, Directive 2003/10/EC [18] sets the exposure limit value at 140 dB. In Polish regulations, 135 dB [19] was adopted as the limit value for exposure. Similarly, the value of the L_EX,8h_ parameter, in accordance with the provisions of the Directive [18], should not exceed 87 dB, while 85 dB was adopted in national regulations [19]. There are also data according to which the A-weighted SPL, which guarantees no impact of noise on hearing even after long-term exposure, is 80 dB [20]. At the same time, L_EX,8h_ at 89 dB can be supplemented since one of the papers concluded that, in the case of impulse noise [21], this was considered a safe value to protect 95% of the population in 95% of cases.

### 2.6. Measuring Equipment

**Figure 2:**
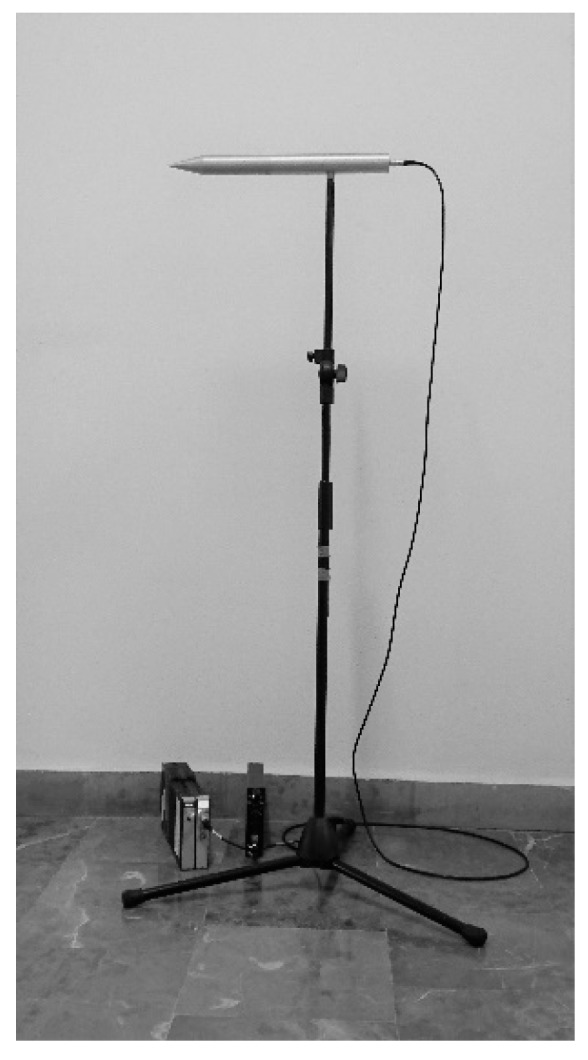
Measuring equipment: G.R.A.S. 67SB transducer on a tripod, bottom left, measuring the cassette of the Brüel & Kjær 3052-A-030 input module (with battery and Wi-Fi modules) and the G.R.A.S. 12AK power module.

In this study, a G.R.A.S. 67SB (GRAS Sound & Vibration A/S, Holte, Denmark) transducer was used for measuring noise parameters, which is designed for impulse noise parameter measurement [14]. The upper limit of the measuring range of this transducer is 174 dB. The electrical signal from the transducer was fed to a G.R.A.S. 12AK power supply module and then to a Brüel & Kjær 3052-A-030 input module (Brüel & Kjær, Nærum, Denmark). A picture of the measuring equipment is shown in Figure 2.

In accordance with the recommendations of EN ISO 9612 [13], the measuring transducer was placed on a tripod at the height of the ear of the shooting instructor. The measurement path was checked using a G.R.A.S. 42AP (GRAS Sound & Vibration A/S, Holte, Denmark) pistonphone. Brüel & Kjær Pulse Reflex (Brüel & Kjær, Nærum, Denmark) software was used to analyze the measurement data.

### 2.7. Methods for the Selection of Hearing Protectors

The assessment of noise reduction by the hearing protectors included in the analysis was carried out by the so-called selection of hearing protectors. The selection consists of calculating the A-weighted SPL $L_{A}^{\prime }$ and the C-weighted peak SPL $L_{\mathrm{Cpeak}}^{\prime }$ under the hearing protectors. For the $L_{A}^{\prime }$ parameter, the selection methods presented in EN 458 [22] were used: the octave band method, the HML method and the SNR method. The results of the octave band method were considered as the reference, because this method is considered to be the most accurate [22]. The other two methods (HML and SNR) were used for comparative purposes. In the case of the $L_{\mathrm{Cpeak}}^{\prime }$ parameter, the methodology for assessing impulse noise reduction is included in informative Annex B of the mentioned standard [22] and is entitled “method for assessing the sound attenuation of the hearing protector for impulse noise”. In view of the fact that impulse noise is generated at the shooting range, level-dependent hearing protectors are then considered as if they were used in passive mode, because the electronic circuits of such protectors block the transmission of sound in the presence of an acoustic impulse.

Calculations with the octave band method are made using sound attenuation data (Table 1) in seven frequency bands ranging from 125 Hz to 8000 Hz. The HML method is based on a smaller number of data and three parameters are used, i.e., H, M and L (introduced in Table 1). The SNR method uses only one parameter (SNR—Table 1), which characterizes the properties of the hearing protector.

In the case of exposure to impulse noise, the assessment of noise reduction by hearing protectors is more complicated, because the instructions for the use of the hearing protector do not contain data related directly to impulse noise. Therefore, appropriately adjusted hearing protector data should be used; i.e., the previously mentioned H or M or L parameters. Due to the fact that a specific hearing protector limits the acoustic impulses generated by various sources to various degrees, these sources are divided into three groups (so-called types of noise), and the method of calculation depends on which type of source is considered. The assignment of the noise source to the noise type is related to the frequency range in which the dominant spectral components of the signal energy generated by the considered source are located. The impulse noise produced during shots from a pistol or a submachine gun is classified as type 3 noise. Then, the value of the $L_{\mathrm{Cpeak}}^{\prime }$ parameter with the hearing protector is calculated from Equation (2). In turn, shots from a smooth-bore shotgun are a source of noise classified as type 2 noise, and then Equation (3) is used. L_Cpeak_ in Equations (2) and (3) is the C-weighted peak SPL measured at the workplace.

(2) $$L_{\mathrm{Cpeak}}^{\prime }=L_{\mathrm{Cpeak}}-H$$

(3) $$L_{\mathrm{Cpeak}}^{\prime }=L_{\mathrm{Cpeak}}-\left(M-5\right)$$

### 2.8. Criteria for the Assessment of Hearing Protectors

The assessment of hearing protectors for their user’s hearing protection is based on a comparison of the calculated values $L_{A}^{\prime }$ and $L_{\mathrm{Cpeak}}^{\prime }$ with the relevant national regulation level, which is specified in the standard for the selection of hearing protectors [22]. Therefore, according to Polish regulations [19], which are more restrictive than the requirements set out in the Directive [18], the $L_{\mathrm{Cpeak}}^{\prime }$ parameter may not exceed the exposure limit value of 135 dB. However, for the selection of hearing protectors in accordance with the $L_{A}^{\prime }$ parameter, the value should be 80 dB. This is a lower exposure action value than that established in Polish regulations and at the same time in Directive 2003/10/EC [18], after which hearing protectors must be provided to an employee. In addition, this is a value that, according to the previously mentioned ISO standard [20], does not result in hearing loss even after long-term exposure to noise. Ultimately, the hearing protector can be considered suitable for hearing protection against noise if the two conditions are met simultaneously; i.e., the $L_{\mathrm{Cpeak}}^{\prime }$ value should not exceed 135 dB, and at the same time, the $L_{A}^{\prime }$ value should not exceed 80 dB.

### 2.9. Statistical Analysis

Statistical analysis of the obtained measurement data was carried out in order to assess whether there were significant differences in exposure to noise resulting from the two variants of the number of shooters simultaneously participating in the exercises and to assess the impact of distance on the values of noise parameters. Data were analyzed using MATLAB R2017b (version 9.3) software with the Statistics and Machine Learning Toolbox (MathWorks Inc., Natick, MA, USA). The analysis used the Wilcoxon test, equivalent to the Mann-Whitney U test.

## 3. Results

### 3.1. Firearm-Related Noise

**Figure 3:**
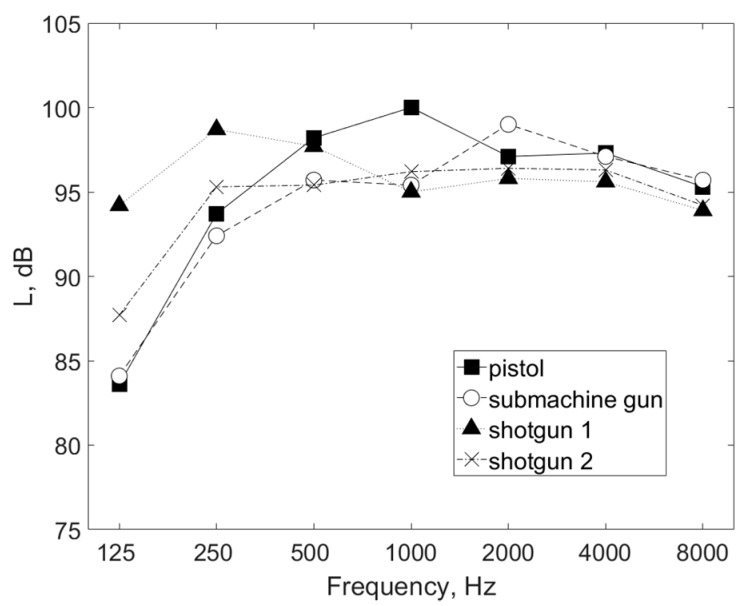
Sound pressure level (SPL) in octave bands representative of one shooting cycle, measured at the shooting instructor’s location at a distance of 1.1 m from six shooters simultaneously taking part in the exercises. The values were determined on the basis of all data obtained during the tests at the shooting range.

Figure 3 shows the noise spectra, i.e., SPL in octave bands values, measured at a distance of 1.1 m from the shooters. Spectral components generally assume values ranging from 84 dB to 100 dB, and the spectrum of the four considered weapon/ammunition combinations as a function of frequency is uneven. The dominant components of acoustic energy associated with pistols are in the 1 kHz band. In the case of the submachine gun, the maximum in the noise spectrum is in the 2 kHz band. Shots from shotgun 1 lead to the spectrum of noise with the highest signal energy in the low-frequency range out of the four weapon/ammunition combinations considered. In the case of shotgun 2, the SPL values in the two lowest frequency bands are 4 dB to 6 dB lower than in the previously mentioned situation (shotgun 1), but still exceed the values measured for pistols and the submachine gun.

**Figure 4:**
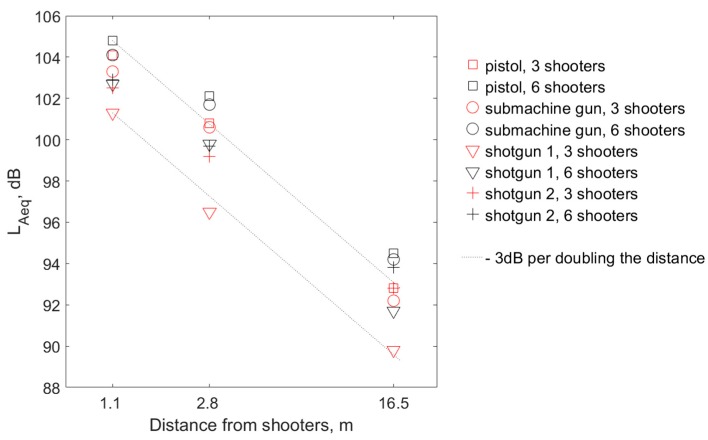
A-weighted equivalent SPL measured at the shooting instructor’s locations, representative of one shooting cycle for both variants of the number of shooters (three or six) simultaneously firing at three different distances between the shooting instructor and the shooters. The values were determined as a result of averaging of all data obtained during the tests at the shooting range under the determined conditions. A logarithmic (base 10) scale is used for the X-axis. Dotted line—a line with a slope of −3 dB per doubling the distance.

Figure 4 shows the L_Aeq_ values measured for both variants of the number of shooters simultaneously shooting, with three different distances between the shooting instructor and the shooters. The highest L_Aeq_ values occur during pistol shots, and the lowest during shotgun 1 shots. The results of the measurements indicate a noticeable decrease in the L_Aeq_ value, associated with the increase in the distance of the instructor from the shooters. The decrease is about 3 dB if the distance is changed from 1.1 m to 2.8 m, and another 8 dB if the distance is increased to 16.5 m. However, the L_Aeq_ values shown in Figure 4 do not fit perfectly in straight lines with a slope of −3 dB per doubling the distance, which is the theoretical slope for free field conditions. This is due to the fact that exercises were held at an indoor shooting range, which is associated with the occurrence of reflections of acoustic waves from the walls and ceiling, which has an impact on the noise parameters. However, it should be noted that the deviations of results from the theoretical line are relatively small, which results from the use of acoustic treatment at the shooting range.

**Figure 5:**
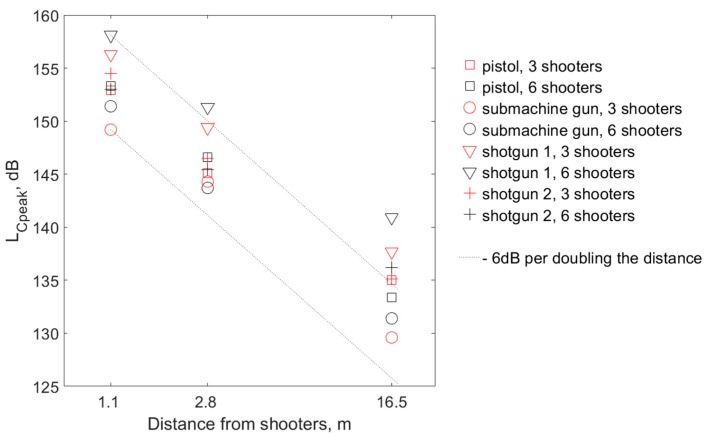
C-weighted peak SPL measured at the shooting instructor’s locations, representative of one shooting cycle for both variants of the number of shooters (three or six) simultaneously firing with three different distances between the shooting instructor and the shooters. The values were determined as a result of averaging all data obtained during the tests at the shooting range under the determined conditions. A logarithmic (base 10) scale is used for the X-axis. Dotted line—a line with a slope of −6 dB per doubling the distance.

In the case of the L_Cpeak_ parameter (Figure 5), fewer shooters are not always associated with a smaller value of this parameter, as was the case for the L_Aeq_ parameter. However, if at some distance of the shooting instructor from the shooters, a certain type of weapon/ammunition combination was associated with a higher value of L_Cpeak_ than the other weapon/ammunition combinations, it was also similar at other distances. It should also be emphasized that the highest L_Cpeak_ values are related to shots from shotgun 1, and the smallest are related to shots from the submachine gun, which is different from the situation regarding the L_Aeq_ parameter. This may be due to differences in the noise spectrum produced by different sources and the filtering of low-frequency components when calculating the L_Aeq_ value.

Similar to the L_Aeq_ parameter, increasing the distance from the shooters was associated with a decrease in the L_Cpeak_ value at the shooting instructor’s location, which does not fit perfectly in straight lines representing the theoretical changes in free field conditions (−6 dB per doubling the distance); however, the deviations of results from the theoretical line are also relatively small (as in the case of L_Aeq_ parameter).

### 3.2. Impact of the Number of Shooters

**Figure 6:**
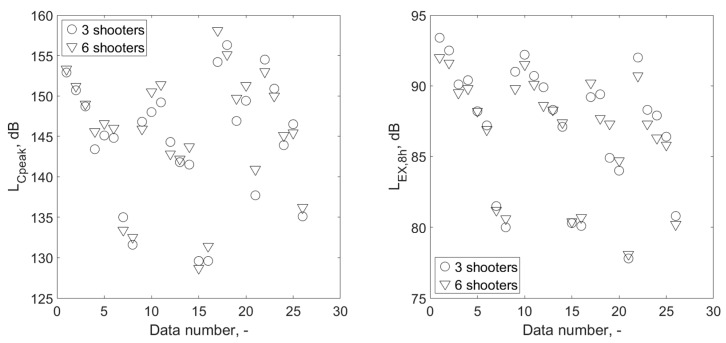
A comparison of L_Cpeak_ and L_EX,8h_ parameter pairs, where each pair of results includes a variant of simultaneous shooting by three and by six shooters, under the same measurement conditions, i.e., at a specified distance from shooters, using a specific weapon/ammunition combination.

The influence of two different, practically applicable scenarios of conducting shooting exercises on the exposure of a shooting instructor to noise was analyzed. Usually, the task of the instructor is to carry out training with a group of 18 shooters in one day. The training may be conducted with a group divided into six three-person subgroups or three six-person subgroups, within which the shooters shoot simultaneously. In the case of three-person teams, the total time of exposure of the instructor to the noise is 1800 s (6 × 300 s), or 1260 s for six-person teams (3 × 420 s). In Figure 6, the L_EX,8h_ values are determined using Equation (1), taking into account the calculated time of exposure to noise, in the case of both variants of the division of shooters into teams. L_Cpeak_ values measured in analogous situations are also included.

In the case of L_EX,8h_, the variation of the value of this parameter related to a different number of shooters is not significant, and the average difference between the situation of three shooters firing in six subgroups and the situation of six shooters in three subgroups is 0.3 dB. It is worth noting that in most cases (16 out of 26), L_EX,8h_ had a higher value for three-person teams than for teams of six people. The reverse situation concerned eight out of 26 cases. The value of L_Cpeak_ associated with the simultaneous firing of shots by three shooters in six subgroups in relation to the situation in which shooting takes place in three subgroups of six shooters, which is more negative than positive (18 and 8 cases out of 26 situations, respectively). On average, the difference in the L_Cpeak_ value between the two variants (three and six shooters) of the number of shooting teams is −0.8 dB.

On the basis of the results of the statistical analysis, it can be concluded that, both in the case of L_Cpeak_ and L_EX,8h_, there are no statistically significant differences between the results obtained with 3-person teams, compared to the results for 6-person teams (*p* = 0.65 and *p* = 0.67 for L_Cpeak_ and L_EX,8h_ parameters, respectively, Wilcoxon test). The conclusion which follows from the described analyses is that a different number of people simultaneously shooting (three or six) does not significantly affect the shooting instructor’s exposure to noise.

### 3.3. Impact of Changing the Distance from Shooters

**Figure 7:**
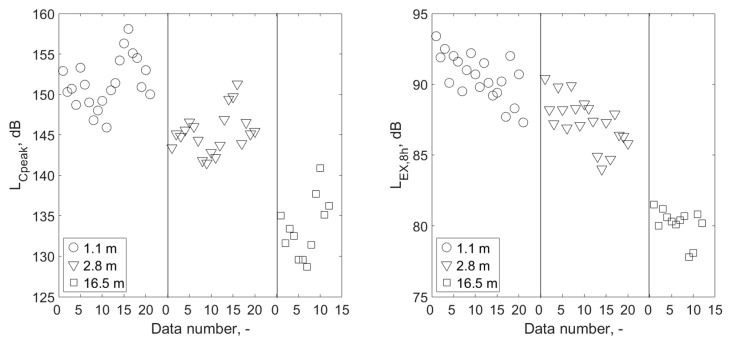
Values of L_Cpeak_ and L_EX,8h_ parameters measured at the shooting instructor’s locations, divided into three variants of the instructor’s distance from the shooters, covering all the types of weapon/ammunition combinations included in the training plan on the shooting range.

The positive effect of increasing the distance of the instructor from the shooters in order to reduce the exposure to noise is basically obvious. However, a verification was made to determine whether, considering the plan of exercises carried out at the shooting range, including the four weapons/ammunition combinations considered, the effects of increasing the distance of the instructor from the shooters should be treated as statistically significant. This verification was carried out with consideration of the parameters used in the noise exposure assessment criteria at the workplace. Figure 7 presents the results for the L_Cpeak_ and L_EX,8h_ parameters, divided into three variants of the instructor’s distance from the shooters. Determined *p*-values for comparisons of the L_Cpeak_ and L_EX,8h_ values corresponding to different distances of the shooting instructor from the shooters; i.e., for the comparison of results measured at 1.1 m and 2.8 m, at distances of 2.8 m, 16.5 m, 1.1 m and 16.5 m, were many times smaller than 0.05 in every situation compared. The results of measurements of noise parameters at different distances were therefore significantly different for individual distances. It has been confirmed, therefore, that the change in distance significantly affects the exposure of the shooting instructor to noise, due to both the L_Cpeak_ and the L_EX,8h_ parameters.

### 3.4. Assessment of Hearing Protectors

**Figure 8:**
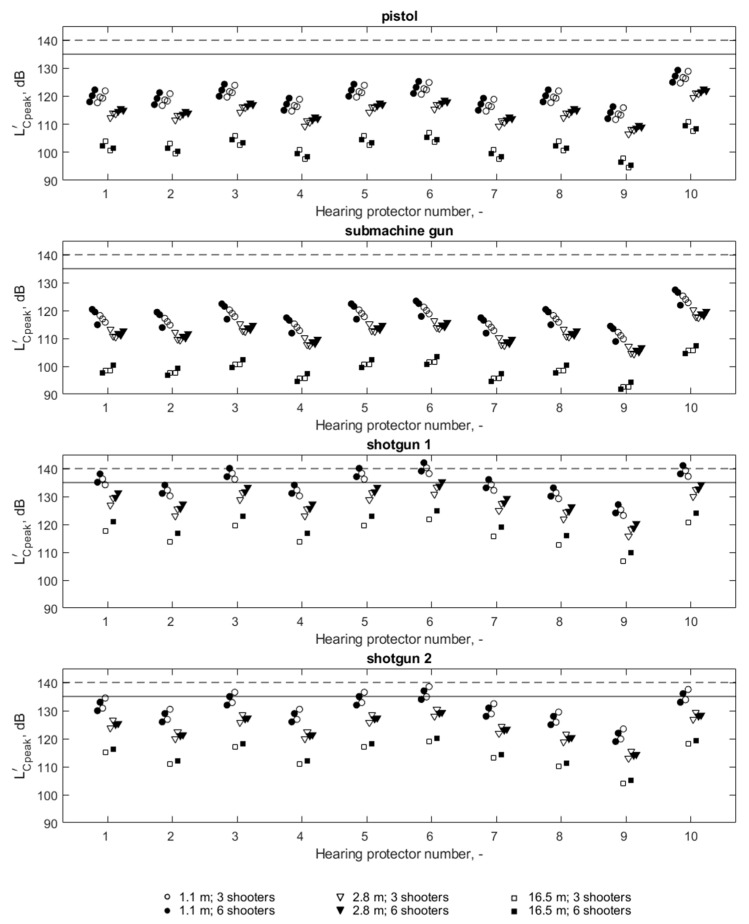
$L_{\mathrm{Cpeak}}^{\prime }$ values (under hearing protectors) for four weapon/ammunition combinations. The horizontal continuous line indicates the criterion value of 135 dB, while the dotted line represents a criterion value of 140 dB.

The results of the selection of hearing protectors with respect to the noise parameter associated with its impulse character, i.e., $L_{\mathrm{Cpeak}}^{\prime }$, are shown in Figure 8. It can be observed that all level-dependent hearing protectors included in the tests make it possible to limit the $L_{\mathrm{Cpeak}}^{\prime }$ value below the criterion value of 135 dB for shots from pistols or a submachine gun. However, for shots from the Mossberg shotgun, the use of some hearing protectors does not result in a sufficient reduction of the $L_{\mathrm{Cpeak}}^{\prime }$ value. For shotgun 2, the $L_{\mathrm{Cpeak}}^{\prime }$ value under four out of 10 hearing protectors exceeds 135 dB, while in the case of shotgun 1, this occurs under six out of 10 protectors. It can also be noted that in the case of the 140 dB criterion, this criterion is exceeded only for shots from shotgun 1, and this takes place for four out of 10 hearing protectors.

**Figure 9:**
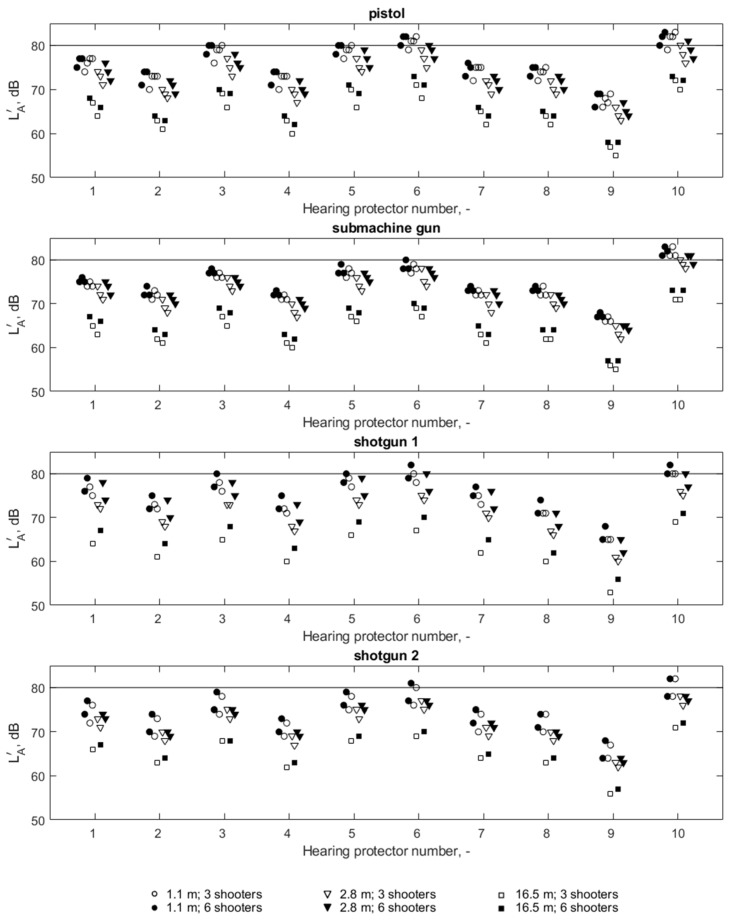
$L_{A}^{\prime }$ values (under hearing protectors) for four weapon/ammunition combinations. A horizontal solid line indicates a criterion value of 80 dB.

The selection of hearing protectors by the octave band method results in the obtained $L_{A}^{\prime }$ values, as shown in Figure 9. The calculations demonstrate that for each of the four weapon/ammunition combinations, not all of the hearing protectors in question resulted in a sufficient reduction of the $L_{A}^{\prime }$ value. Hearing protector number 10 did not fulfil its task in relation to all four weapon/ammunition combinations, while hearing protector number 6 sufficiently limited the $L_{A}^{\prime }$ value only in the case of the submachine gun.

The highest absolute values of difference between the calculations of the $L_{A}^{\prime }$ value using the octave band method and the HML method were 2 dB, while between the octave band and the SNR method, the difference reached 4 dB. The results of the comparison with the criterion value of $L_{A}^{\prime }$, calculated using the SNR method and the octave band method, were not consistent in individual measurement situations; however, the SNR method ultimately ruled out the same two protectors from the group of appropriate hearing protectors with the octave band method. The result is slightly different when comparing the results of using the HML method and the octave band method, where the HML method excluded four hearing protectors (marked as 3, 5, 6 and 10), while according to the results of the octave band method, an inadequate reduction of $L_{A}^{\prime }$ values took place in the case of two hearing protectors (6 and 10). The results obtained indicate, therefore, that if suitable measurement data are available, the most accurate selection method should be used: i.e., the octave band method. The use of other methods for calculating the $L_{A}^{\prime }$ value may result in different conclusions from the octave band method, which is considered as the reference method.

The highest values of $L_{\mathrm{Cpeak}}^{\prime }$ and $L_{A}^{\prime }$ determined from all the measuring situations included in the study are shown in Table 2.

## 4. Discussion

The research conducted indicated that the noise produced in individual training situations at the shooting range was different in terms of the content of acoustic energy components in individual frequency bands (Figure 3). Taking this fact into account, and taking into consideration that the characteristics of the sound attenuation of hearing protectors as a function of frequency are uneven (Table 1), it should be stated that the assessment of the reduction of the noise produced at a shooting range by individual hearing protectors requires appropriate calculations in each situation considered.

The measurements of the impulse noise parameters generated in the places where the shooting instructor was located indicated relatively high SPL values produced by the firearms, which is in line with the results of other studies on shots from firearms. Measurements conducted in this study showed that the instructor, when standing at a distance of 1.1 m behind the shooters, was exposed to acoustic impulses whose L_Cpeak_, depending on the variant of weapon/ammunition combination and the shooting scenario, assumed values ranging from 145.9 dB to 158.1 dB. There are, however, no data that could be directly compared with the data obtained in this study at the indoor shooting range for the instructor’s locations. Published results usually characterize noise close to the shooter’s ear (although not always), or there is no precise information about the measurement location and C-weighted peak SPL or unweighted peak SPL (L_peak_) being considered; moreover, measurements are usually carried out on military fields. For example, in [1], there is general information that the peak SPL produced by small-caliber rifles, shotguns and large-caliber handguns ranged from 132 dB up to more than 172 dB for high-powered firearms. In the study in which the measurements were carried out in field conditions, a few meters away from the shooter, the L_peak_ associated with C7 rifle shots was 148.3 dB, while at the distance of 30 cm from the shooter, it was 154.7 dB [23]. For shots from a 9 mm caliber pistol, values of 148.4 dB and 155.6 dB were obtained, respectively [23]. Subsequent data [24] indicated that for L_peak_ determined on the basis of waveforms associated with a 5.56 mm caliber C7 rifle shot, recorded at a distance of 4 m from the muzzle at 90° and 6.4 m at 39°, in both cases, the value was approximately 157 dB. In one of the studies, the L_peak_ values associated with AR-15 rifle shots were 168 dB at the distance of 1.8 m, 150 dB at 4.3 m, and 132 dB at 25.7 m [25]. Despite the mentioned differences between the aforementioned studies and this study regarding the measurement conditions, the common point is that large and comparable SPLs are measured in the places where the shooting instructor is located. The exposure of the shooting instructor to the noise produced at the shooting range, which, according to the previously mentioned criteria should be classified as dangerous for hearing, requires the use of appropriately selected hearing protectors. Another conclusion is that in order to correctly determine the values of the noise parameters to obtain the necessary data for the selection of hearing protectors, the measurements could not be carried out using standard sound level meters. The upper measuring range of such devices is usually limited to approximately 145 dB. In response to the problem of measuring high SPL values related to impulse noise, hardware solutions were developed [14,26].

In one of the studies, where strategies for possible ways to reduce the negative impact of impulse noise on shooters’ ears were formulated, among others, the use of outdoor or acoustic-treated indoor shooting ranges was recommended [27]. The results obtained on the shooting range included in the present study confirmed that when applying acoustic treatment, acoustic conditions similar to the free field conditions can be obtained. Although the L_Aeq_ and L_Cpeak_ values shown in Figure 4 and Figure 5 do not fit perfectly in straight lines with the theoretical slope for free field conditions, the deviations of results from the theoretical lines are relatively small. This proves that the use of acoustic treatment on the shooting range effectively limited the impact of sound reflections. Therefore, it can be assumed that the effect of the distance of the shooting instructor from the sound source on the degree of hearing hazard is similar to the situation in free field conditions.

In the case of impulse noise, it is not possible to characterize the reduction of this kind of noise by a specific hearing protector using one value only. This is due to the fact that the parameters of acoustic impulses produced by different sources differ from one another [22], and the reduction of the impulse noise strongly depends on the source of this noise [25]. According to the methodology for hearing protector assessment referring to the L_Cpeak_ parameter, in this paper, for each hearing protector, two attenuation values were distinguished: one for shots from pistols and a submachine gun, and the second for shots from smooth-bore shotguns (shotgun 1 and shotgun 2). The different values of noise parameters produced by each weapon/ammunition combination and the different values of the acoustic parameters of individual hearing protectors resulted in different noise values determined under the hearing protectors. For example, the highest $L_{\mathrm{Cpeak}}^{\prime }$ values (Figure 8) under the HP1 hearing protector were 122.3 dB (pistols), 120.4 dB (submachine gun), 138.1 dB (shotgun 1) and 134.5 dB (shotgun 2). The range of these values is therefore 17.7 dB. Similarly, the range of the $L_{\mathrm{Cpeak}}^{\prime }$ values among the four weapon/ammunition combinations, depending on the hearing protector, ranges from 12.7 to 18.7 dB for all other hearing protectors. At the same time, it should be emphasized that with a particular hearing protector, the $L_{A}^{\prime }$ parameter values are much less varied (from 1 dB to 3 dB—Figure 9) between different weapon/ammunition combinations than they are in the case of the $L_{\mathrm{Cpeak}}^{\prime }$ parameter. Therefore, for impulse noise, the parameter reflecting the energy properties of noise ($L_{A}^{\prime }$) differentiates individual noise sources to a much lesser extent than the parameter referring to the instantaneous values characterizing impulses ($L_{\mathrm{Cpeak}}^{\prime }$). It is therefore confirmed that for exposure to impulse noise, the assessment of hearing protectors is insufficient due to the value of the $L_{A}^{\prime }$ parameter, as is the case for continuous noise. It is necessary to take into account the $L_{\mathrm{Cpeak}}^{\prime }$ parameter, which of course is primarily due to the fact that the L_Cpeak_ value of noise is exceeded.

There are no published data on the reduction of impulse noise by individual hearing protectors, which could have been directly compared with the results presented in this paper. For example, data are available for the insertion loss of impulses generated during shots from an AR-15 rifle with a L_peak_ of 150 dB [25]. These data were measured using an acoustical test fixture for earmuffs with similar properties to the hearing protector designated in this paper as HP2. The earmuff from the aforementioned study [25] reduced the L_peak_ to 108.8 dB. In similar measurement conditions in the next study, data were obtained that indicated that another earmuff reduced L_peak_ to 118 dB [28]. In this study, in the most similar situations to the two mentioned studies, the average L_Cpeak_ value with the HP2 protector at distances from the shooter equal to 1.1 m and 2.8 m was 113.3 dB for submachine gun shots and 125.0 dB for shotgun 2 shots. Examples of published data can be supplemented with the result of 132 dB concerning the L_peak_ parameter measured using an acoustical test fixture with an earmuff, in the presence of an impulse produced during a shot from a 12.7 mm machine gun [29]. The impulses produced by this source were characterized by a L_peak_ value of 152 dB. The studies in the referenced papers [25,28,29] and this paper differ in the type of weapon, noise parameter analyzed, hearing protector model, distance from the shooter and measurement method used; however, independently obtained values of noise parameters potentially reaching the user of the hearing protector exceed 100 dB and are below the exposure limit values. It can be observed that in all the studies discussed, the reduction of impulse noise by hearing protectors was measured using a head and torso simulator; i.e., an acoustical test fixture. This is different from this particular study, in which a calculation method was employed in which sound attenuation data of hearing protectors was used, which are determined with the participation of subjects.

Since a hearing protector can only be considered suitable for protecting hearing against noise when its use obtains correspondingly reduced parameter $L_{\mathrm{Cpeak}}^{\prime }$ and $L_{A}^{\prime }$ values at the same time. On the basis of the data in Table 2, it can be stated that, according to the adopted criteria for the assessment of hearing protectors, $L_{A}^{\prime }\;$ = 80 dB and $L_{\mathrm{Cpeak}}^{\prime }$ = 135 dB, an appropriate hearing protection for the shooting instructor will be possible when using hearing protectors 2, 4, 8 and 9. Two of the earmuffs (hearing protectors 2 and 4) and earplugs in two variants of eartips (hearing protectors 8 and 9) were found to be appropriate.

At the same time, it can be noted that by changing the criterion for $L_{\mathrm{Cpeak}}^{\prime }$ to less severe, i.e., $L_{\mathrm{Cpeak}}^{\prime }$ = 140 dB, as used in many countries and defined in the Directive [18], an adequate protection of hearing would also be guaranteed by hearing protectors 1 and 7.

The results obtained indicated that the parameter associated with the instantaneous value of the signal, i.e., L_Cpeak_, is crucial in the process of assessing hearing protectors in terms of their ability to reduce the impulse noise produced on a shooting range. In both situations in which the parameter $L_{A}^{\prime }$ value was exceeded, the criterion value referring to the parameter $L_{\mathrm{Cpeak}}^{\prime }$ was also exceeded. At the same time, in four consecutive situations of exceeding the criterion value of the $L_{\mathrm{Cpeak}}^{\prime }$ parameter, these situations did not involve exceeding the $L_{A}^{\prime }$ parameter. It is therefore important that, for impulse noise, the selection of hearing protectors cannot be limited to taking into account the $L_{A}^{\prime }$ parameter, as in the case of continuous noise, where there is no problem in exceeding the exposure limit values of L_Cpeak_.

At the same time, it should be noted that hearing protection requires not only properly selected hearing protectors, but that they must be used and set up correctly, and other sources of noise exposure, often unrelated to professional work, should be avoided. For example, practical instances of the improper use of earplugs were observed, and the reason behind this was a lack of training or that it was deliberately done to better hear messages spoken by the Range Safety Officer [23]. In turn, in one of the papers investigating the hearing condition of 20 policemen after 10 years of service, a deterioration of hearing was found despite the use of double hearing protection [30].

## 5. Conclusions

The results of the study indicate that impulse noise produced on the shooting range should be assessed as dangerous for hearing. The exposition of the shooting instructor to this noise requires the use of appropriately selected hearing protectors. The results of the analysis of noise parameters measured at different distances of the shooting instructor from the shooters confirmed that increasing this distance, if possible, is a good method for reducing the exposure of the instructor to noise. It also appeared that with a certain number of shooters who are to be trained, their division into subgroups consisting of three or six people at the same time does not significantly affect the shooting instructor’s exposure to noise.

The results of the assessment of level-dependent hearing protectors demonstrated that it is possible to adequately protect the hearing of a shooting instructor who is at an indoor shooting range when using protectors of this type. It is possible to choose hearing protectors, both earmuffs and earplugs, that will sufficiently reduce the impulse noise to which the instructor is exposed.

It appears that, taking into account the program of exercises at the shooting range at which shooting was carried out with pistols, a submachine gun and smooth-bore shotguns, the assessment of hearing protectors with a more restrictive criterion $(L_{A}^{\prime }$ = 80 dB, $L_{\mathrm{Cpeak}}^{\prime }$ = 135 dB) showed that only four out of the 10 level-dependent hearing protectors included in the analysis sufficiently reduced the noise. Assuming a less restrictive criterion regarding the C-weighted peak SPL, i.e., $L_{\mathrm{Cpeak}}^{\prime }$ = 140 dB, six out of 10 hearing protectors would be appropriate. Therefore, regardless of which criterion for the assessment of earmuffs is used, only some of the hearing protectors will be a potentially suitable means of protecting the shooting instructor’s hearing, and the correct selection of hearing protectors is necessary. However, in the case of impulse noise, this selection must be based not only on the standardized selection methods related to the continuous noise but must also take into account the parameter associated with the instantaneous value of the signal level; i.e., the C-weighted peak SPL.

## Figures and Tables

**Table 1:** Sound attenuation data of level-dependent hearing protectors. m_f_—mean attenuation ^1^, s_f_—standard deviation, H—high-frequency attenuation value ^2^, M—medium-frequency attenuation value, L—low-frequency attenuation value, SNR—single number rating, HP1 to HP10—hearing protectors 1 to 10.

Designation	m_f_/s_f_ [dB]	Frequency [Hz]							H [dB]	M [dB]	L [dB]	SNR [dB]
		125	250	500	1000	2000	4000	8000				
HP1	m_f_	11.5	17.9	27.8	30.0	32.1	36.2	40.3	31 ^3^	25	16	28
	s_f_	2.5	2.7	1.8	2.3	3.0	2.0	3.1				
HP2	m_f_	13.8	21.5	30.9	36.6	35.9	35.5	39.0	32	29	20	31
	s_f_	1.8	0.9	1.3	1.5	5.5	3.1	2.3				
HP3	m_f_	21.1	17.9	27.0	26.8	30.5	38.3	36.4	29	23	16	26
	s_f_	4.3	3.1	3.8	3.0	3.0	3.7	5.4				
HP4	m_f_	17.0	24.0	29.5	36.9	37.3	39.3	35.4	34	29	22	32
	s_f_	3.2	2.0	2.6	3.3	4.9	3.2	3.9				
HP5	m_f_	13.3	17.4	22.3	28	30.8	37.6	37.0	29	23	17	26
	s_f_	3.2	1.8	2.3	3.2	3.4	2.8	4.8				
HP6	m_f_	13.5	15.5	23.7	24.1	30.4	36.6	38.6	28	21	16	25
	s_f_	3.2	1.9	3.9	2.7	3.2	4.4	4.2				
HP7	m_f_	18.4	21.1	27.7	36.9	36.1	42.1	38.8	34	27	20	30
	s_f_	4.5	4.2	3.5	4.0	3.6	3.5	5.4				
HP8	m_f_	34.5	31.5	36.2	33.4	34.8	34.9	38.8	31	30	29	32
	s_f_	6.0	5.4	5.6	4.3	3.8	5.0	4.0				
HP9	m_f_	37.8	36.0	40.5	41.2	41.3	39.6	46.1	37	36	34	38
	s_f_	4.3	5.5	4.2	4.7	3.2	4.3	3.6				
HP10	m_f_	15.7	19.1	22.9	27.0	22.4	38.4	40.9	24	22	18	25
	s_f_	3.0	3.1	2.9	2.3	3.3	3.0	3.4				

^1^ Definitions of m_f_ and s_f_ are given in EN ISO 4869-1 [15]; ^2^ definitions of H, M, L and SNR are given in EN ISO 4869-2 [16] and have since been slightly modified in the current version of this standard [17]; ^3^ values of H, M, L and SNR are rounded to the nearest integer [16,17].

**Table 2:** The highest values of noise parameters under hearing protectors determined from all the measuring situations included in the study (53 shooting cycles).

Hearing Protector Number	1	2	3	4	5	6	7	8	9	10
$L_{\mathrm{Cpeak}}^{\prime }$, dB	**138.1**	134.1	**140.1**	134.1	**140.1**	**142.1**	**136.1**	133.1	127.1	**141.1**
$L_{A}^{\prime }$, dB	79	75	80	75	80	**82** ^1^	77	75	69	**83**

^1^ Results exceeding the relevant criterion value are provided in bold. Criteria values: $L_{A}^{\prime }$ = 80 dB, $L_{\mathrm{Cpeak}}^{\prime }$ = 135 dB.
